# HKD-MGIN: a physics informed graph neural network using heat kernel diffusion for mapping adolescent functional brain connectivity

**DOI:** 10.1007/s44379-025-00038-8

**Published:** 2025-10-20

**Authors:** B. Patel, Z. Habeeb

**Affiliations:** 1 Biomedical Engineering Department, Tulane University, New Orleans, LA, USA; 2 Psychology Department, California State University, Fresno, CA, USA

**Keywords:** Deep learning, Heat kernel diffusion, Graph isomorphism network, fMRI, Interpretable, Multi-task

## Abstract

Adolescence is a critical period characterized by dynamic changes in cognitive function and brain network organization. This study aims to model these complex neurodevelopmental processes using a novel graph-based learning framework that captures both local and global functional interactions across adolescent stages. We introduce the Heat Kernel Diffused Multi-task Graph Isomorphism Network (HKD-MGIN), which integrates multi-task functional magnetic resonance imaging (fMRI) data using heat kernel diffusion. This approach leverages a graph isomorphism network architecture to enable robust and interpretable representation learning. HKD-MGIN is applied to two predictive tasks: brain age estimation and sex classification. HKD-MGIN demonstrated strong performance in brain age estimation (RMSE = 1.864 ± 0.157; MAE = 1.492 ± 0.139; r = 0.816 ± 0.042) and achieved high accuracy in sex classification (ACC = 0.802 ± 0.015; AUC = 0.834 ± 0.017; F1 Score = 0.8199 ± 0.0514). Importantly, the model revealed functional circuits associated with cognitive maturation, highlighting its capacity for interpretability and neurobiological insight. These findings demonstrate the utility of heat kernel-based graph learning in modeling adolescent brain dynamics and identifying potential biomarkers of cognitive development. HKD-MGIN advances neuroimaging analysis by capturing hierarchical and distributed connectivity patterns, offering a promising direction for individualized tracking of neurodevelopmental trajectories.

## 1 Introduction

Adolescence is a critical period of neurodevelopment marked by significant structural and functional changes in the brain, which are crucial for the maturation of cognitive, emotional, and social capabilities. These changes can influence behavior and mental health throughout adulthood, emphasizing the importance of understanding the neurobiological processes underlying this developmental stage [3, 4]. Functional magnetic resonance imaging (fMRI), has emerged as a powerful tool to explore the complex interactions between brain regions, providing insights into both typical and atypical brain development. Functional connectivity measures derived from fMRI data, such as Pearson correlation coefficients, offer a window into the intricate relationships among brain networks [5, 6]. However, analyzing high-dimensional and inter-correlated fMRI data presents significant challenges, necessitating advanced computational methods to extract meaningful insights.

Heat kernel diffusion models have shown promise in brain network analysis, leveraging the principles of heat diffusion to quantify connectivity patterns and capture the underlying topology of functional and structural networks. These methods have been instrumental in predicting functional connectivity from structural connectivity by modeling dynamic processes over multiple timescales [6, 7]. Integrating diffusion-based approaches with multimodal data, such as task-based and resting-state fMRI, has further demonstrated the ability to enhance predictive performance, revealing nuanced brain-behavior relationships and improving the understanding of neurodevelopmental trajectories [3, 4]. Despite these advances, traditional heat diffusion models often face limitations in scalability and computational efficiency, particularly when applied to high-dimensional data from large-scale neuroimaging studies [5, 9].

Recent developments in graph-based machine learning, such as Graph Neural Networks (GNNs), have opened new avenues for modeling brain connectivity. By leveraging graph structures, these methods provide a powerful framework for integrating multimodal neuroimaging data while preserving the geometric and topological properties of brain networks. The heat kernel diffusion graph approach has emerged as a promising technique in this domain, enabling the exploration of complex interactions across different modalities and time scales [6, 7]. Combining heat kernel diffusion with multimodal graph isomorphism networks offers the potential to improve the representation of brain connectivity, address challenges related to dimensionality and noise, and facilitate the identification of biomarkers for neurodevelopmental and neuropsychiatric conditions [4, 9].

In this study, we propose a Heat Kernel Diffusion Graph Isomorphism Network (HKD-MGIN) to enhance the analysis of brain connectivity patterns during adolescence. Our approach integrates heat kernel diffusion processes with graph isomorphism techniques, allowing for the simultaneous analysis of structural and functional data. By capturing the dynamic and multiscale nature of brain networks, the proposed method aims to provide deeper insights into typical neurodevelopment and neurodivergence. Furthermore, the HKD-MGIN framework addresses computational challenges by incorporating efficient numerical approximations and scalable algorithms, making it well-suited for large-scale neuroimaging datasets.

Our method offers three key contributions. First, in terms of biological relevance, the heat kernel naturally aligns with the brain’s hierarchical and distributed architecture, making it well-suited for capturing both localized and large-scale patterns of neural activity. Second, the approach enhances the expressiveness of network representations by modeling how information flows across the brain, analogous to the diffusion of heat through a graph. This is particularly valuable for tasks such as decoding functional connectivity, predicting brain age, or identifying neurobiological biomarkers. Finally, the framework captures both local and global connectivity patterns, offering a more comprehensive view of brain network dynamics compared to traditional random-walk diffusion methods, which primarily focus on local interactions.

The rest of the manuscript is organized as follows: Section 2 reviews related work, Section 3 introduces the HKD-MGIN framework, Section 4 presents experimental results and ablation studies, Section 5 discusses neurodevelopmental insights, and Section 6 concludes the paper with limitations and future directions.

## 2 Related work

Graph and diffusion models have become powerful tools in brain imaging, causal learning, and understanding brain networks. One important development is multi-view graph models, which combine different types of brain data and help improve predictions across tasks. For example, Yu et al. [1] used a multi-view graph with similarity diffusion to achieve top results in zero-shot learning. However, using these advanced models in brain imaging is still difficult because of the complexity of combining multiple types of brain data and bridging the gap between how the brain is built and how it functions.

Although diffusion-based methods are useful in areas like network modeling, they face challenges in brain studies. Karimi Mamaghan et al. [2] created a method called Diffusion-based Causal Representation Learning, which finds hidden causal patterns in both small and large data. But using this with noisy and high-dimensional brain imaging data is hard. Similarly, Pan et al. [3] used multi-view diffusion maps to combine resting-state and task-based fMRI, which helped predict intelligence levels. Although this shows the power of diffusion for combining data types, problems like scaling to larger datasets, handling noise, and adapting to specific tasks remain.

Heat kernel based models have been especially good at mapping the shape and connections of brain networks. Chung et al. [4] showed that the characteristics of the heat kernel can tell us about network efficiency and predict motor outcomes in preterm infants. To make things faster, Huang et al. [5] developed a quick polynomial method for approximating heat diffusion, which helped make large brain graphs more manageable. Still, heat kernel methods can be computationally expensive, especially for big or high-resolution fMRI data.

Some researchers are tackling these issues by combining heat diffusion with deep learning. Lv et al. [6] created a model that predicts brain function based on structure by using heat diffusion on different time scales. This shows how important time-based modeling is, but also increases the complexity of the model and requires a lot of data.

Efforts to connect structural and functional brain data have also used attention-based and multi-scale methods. Oota et al. [7] built a model called GraphHeat that uses attention and heat diffusion to connect brain structure to function. Although promising, these methods often require large datasets and fine-tuned parameters, which may not work well for smaller or noisier data. In a different approach, Chung et al. [8] combined diffusion wavelets and kernel regression to study age-related brain changes, especially in limbic areas. This method mixed geometric and statistical tools well, but questions remain about how well it works across different tasks or groups. Other work shows that functional diffusion maps can track how the brain responds to treatment. For example, Ruiz-Espaa et al. [9] used diffusion MRI to study brain metastases during therapy. Although the results are promising, applying this more widely is still difficult due to challenges in combining different data types and aligning them over time.

Despite progress, key challenges remain in understanding brain networks. Brain connections work on many levels, and good models need to capture local and global activity without being overwhelmed by noise or becoming too complex. Traditional graph methods can struggle with smoothing too much, being difficult to interpret biologically, or missing long-range brain connections. This is where heat-kernel diffusion can help: it uses ideas from physics, modeling how information flows through a network like heat spreading across a surface. This helps capture both nearby and faraway brain connections in a more natural way. However, using this in practice is still hard due to limitations in computation and how it fits with newer deep learning tools. Our approach aims to solve these problems by combining the mathematical strengths of heat diffusion with the power of graph isomorphism networks. This helps us better analyze complex fMRI data from tasks and resting states alike.

## 3 Methodology

### 3.1 Overview of pipeline

**Fig. 1:**
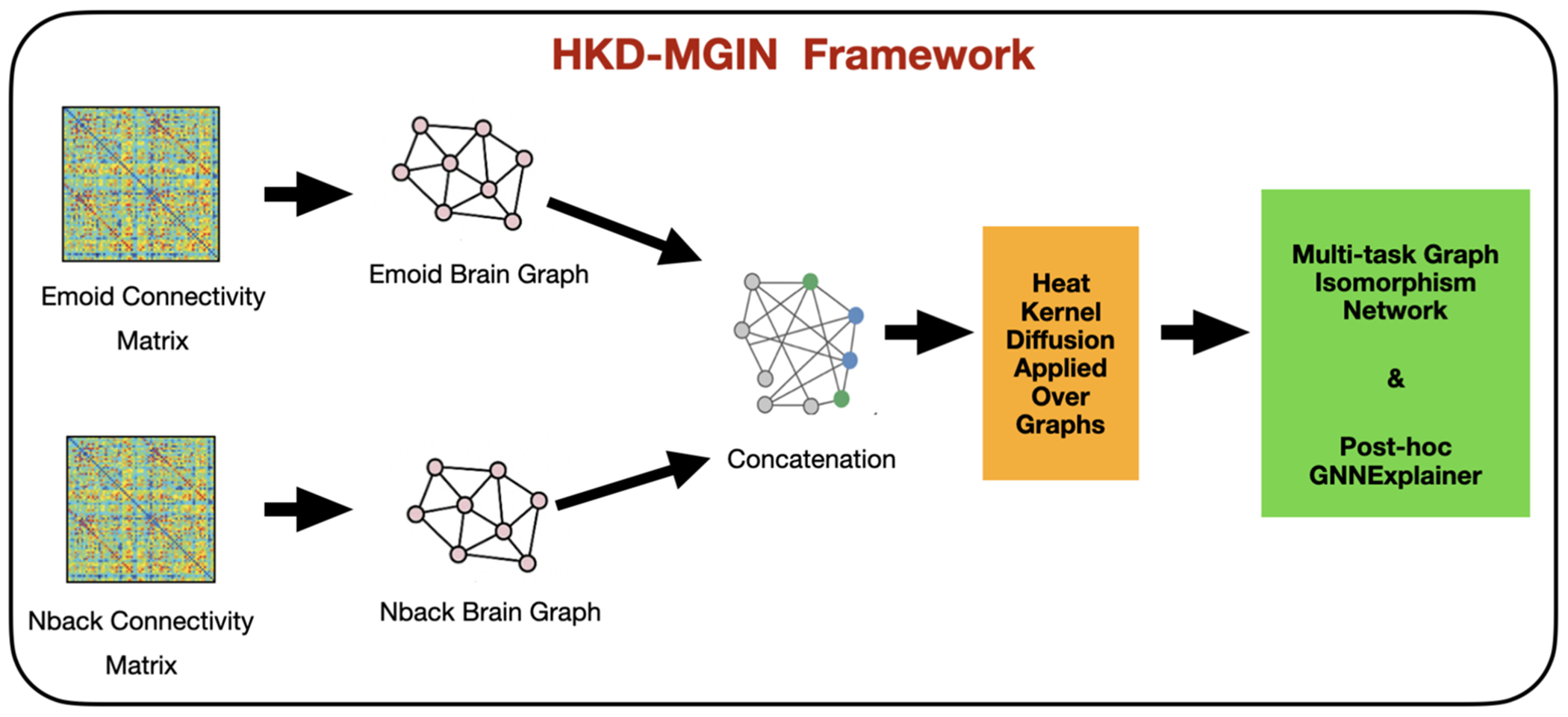
Schematic overview of the HKD-MGIN processing pipeline. fMRI data from emoid and nback paradigms undergo preprocessing and parcellation using the Power atlas (264 ROIs). Functional connectivity matrices are computed via Pearson correlation, processed through heat kernel diffusion, and analyzed using graph isomorphism networks for age prediction and sex classification

The methodological framework of this investigation is illustrated in Fig. 1. The protocol commenced with pre-processing of individual participants’ functional magnetic resonance imaging (fMRI) temporal sequences. Subsequently, whole-brain parcellation was performed utilizing the Power atlas framework [10], yielding 264 discrete regions of interest (ROIs). For each ROI, investigators extracted the mean Blood Oxygen Level Dependent (BOLD) signal trajectory and computed inter-regional Pearson correlation coefficients to generate functional connectivity matrices. The resulting brain network representations, derived from both emotional processing (emoid) and working memory (nback) paradigms, were concatenated. This unified dataset was then analyzed using our novel Heat Kernel Diffused Multimodal Graph Isomorphism Network architecture to facilitate age-based prediction and sex-based classification across adolescent developmental stages (Table 1). The analysis culminated in the application of GNNExplainer methodology to elucidate crucial subnetwork architectures and identify salient functional connections within the neural networks [11].

### 3.2 Data collection and pre-processing

The dataset used in this study originates from the Philadelphia Neurodevelopmental Cohort (PNC), a collaborative initiative between the Children’s Hospital of Philadelphia and the University of Pennsylvania, funded by the National Institute of Mental Health (NIMH). The PNC aims to investigate the interplay between brain function and behavior, encompassing data from over 800 healthy participants aged 8 to 22 years. This study utilized functional MRI (fMRI) data from two task paradigms: emoid and nback. All imaging was performed on a 3T Siemens TIM Trio whole-body scanner. During the emoid task, participants categorized facial expressions into emotional categories, including happiness, anger, sadness, fear, and neutrality. Scans were conducted over a 10.5-minute session, capturing the blood oxygen level-dependent (BOLD) signal with a whole-brain, single-shot, multislice, gradient-echo echoplanar imaging sequence comprising 124 volumes [12].

Preprocessing of the imaging data was carried out using SPM12, including steps such as motion correction, spatial normalization to the standard MNI template, and spatial smoothing using a Gaussian kernel with a full width at half maximum of 3mm [12]. The Power template [10] was employed to parcellate the brain into 264 regions of interest (ROIs), facilitating the extraction of BOLD signals and the construction of connectivity matrices via Pearson correlation. For this study, 622 participants were selected from the full cohort, based on the availability of both emoid and nback task data. These participants were further stratified into five distinct developmental stages representing key phases of adolescence [13].

### 3.3 Heat kernel diffusion

Heat kernel diffusion is a sophisticated mathematical technique that has recently gained traction in the analysis of fMRI data, particularly for capturing the underlying geometry of functional brain networks. The heat kernel serves as a fundamental solution to the heat equation on a graph, facilitating the propagation of information across the network structure. This approach is well-suited for fMRI data analysis because it leverages the connectivity patterns of the brain and encodes the diffusion process, effectively capturing the intrinsic relationships between brain regions over time.

Consider an undirected graph 𝒢=(𝒱,ℰ), where 𝒱 represents the set of *N* brain regions (nodes), and ℰ denotes the edges representing functional or structural connections. The graph can be characterized by its adjacency matrix $A\in R^{N\times N}$ and its degree matrix **D**, where $D_{ii}=\sum_{j}A_{ij}$. The Laplacian matrix **L** = **D** – **A** encodes the graph’s topology. The heat kernel matrix **K***_t_* at time *t* is defined as:

(1) $$K_{t}=e^{-tL},$$

where *t* is the diffusion time parameter and *e*^−*t***L**^ is the matrix exponential of the negative Laplacian. The heat kernel matrix **K***_t_* can be interpreted as capturing the amount of heat (or information) diffused between each pair of nodes over time *t*. It provides a measure of the connectivity strength between nodes, accounting for both direct and indirect interactions within the graph.

In the context of fMRI data, the heat kernel diffusion process can be used to generate a diffused functional connectivity matrix, which reflects the smoothed interaction patterns across brain regions. Given a fMRI time series matrix $X\in R^{N\times M}$, where *M* is the number of time points, the diffused signal matrix **X***_t_* can be obtained as:

(2) $$X_{t}=K_{t}X.$$

This transformation enhances the signal by incorporating the structural information encoded in the heat kernel, effectively smoothing the time series data based on the graph topology. The choice of *t* controls the scale of diffusion: smaller values of *t* emphasize local connectivity, while larger values capture more global interactions across the brain network.

The objective of incorporating heat kernel diffusion in fMRI analysis is to improve the identification of functional connectivity patterns and brain network organization. By smoothing the time series data through diffusion, this method reduces noise and highlights coherent connectivity structures that are not immediately evident in the raw fMRI signals. It can be particularly beneficial for analyzing resting-state fMRI data, where the goal is to detect intrinsic connectivity networks (ICNs) that represent consistent patterns of brain activity.

Moreover, heat kernel diffusion has been applied in the context of brain age prediction and neurodevelopmental studies, providing a robust framework for assessing the maturation of functional brain networks. The smoothed connectivity patterns extracted via heat kernel diffusion are used as features for predictive modeling tasks, such as classifying clinical populations or estimating brain age in adolescence. This approach captures the multiscale nature of brain connectivity and provides a more comprehensive view of the brain’s functional architecture [14–16]. By leveraging the graph’s structural information, heat kernel diffusion offers a powerful tool for analyzing fMRI data, enhancing the ability to detect meaningful patterns of brain activity and connectivity in both healthy and clinical populations.

### 3.4 Graph Isomorphism Network (GIN)

The Graph Isomorphism Network (GIN) is a specialized type of spatial Graph Neural Network (GNN) designed for tasks involving graph classification. Initially introduced by *Xu et al.*, GIN aims to effectively capture the topological structure of input graphs while learning powerful node representations through a process of aggregation and combination of node features [17]. The key innovation of GIN lies in its design, which closely aligns with the Weisfeiler-Lehman (WL) test for graph isomorphism, making it one of the most expressive GNN architectures for distinguishing non-isomorphic graphs.

GIN updates node embeddings by iteratively applying an aggregation function followed by a combination function, as shown in Eq. 3. The update rule for the node feature vector at the *k-th* layer is defined as:

(3) $$h_{v}^{\left(k\right)}={\text{MLP}}^{\left(k\right)}\left(\left(1+\epsilon^{\left(k\right)}\right)\cdot h_{v}^{\left(k-1\right)}+\underset{u\in 𝒩(v)}{\sum }h_{u}^{\left(k-1\right)}\right),$$

In this equation, $h_{v}^{\left(k\right)}$ is the feature vector of node *v* at the *k-th* layer, while $h_{v}^{\left(k-1\right)}$ represents the feature vector from the previous layer. The parameter *ϵ^(k)^* is a learnable scalar that allows the model to adaptively weigh the importance of the node’s own features. The multi-layer perceptron (MLP) applies a non-linear transformation, enabling the model to capture complex feature interactions across nodes. The aggregation step sums the features of neighboring nodes, while the combination step integrates these aggregated features with the node’s own features.

To obtain a graph-level representation, GIN employs a readout function that aggregates node embeddings from each layer. The graph-level embedding $h_{G}^{\left(k\right)}$ at the *k-th* layer is computed by summing the node embeddings, as shown in Eq. 4:

(4) $$h_{G}^{\left(k\right)}=\text{sum}\left(h_{0}^{\left(k\right)},h_{1}^{\left(k\right)},\ldots ,h_{N}^{\left(k\right)}\right),$$

The final graph embedding *h_G_* is obtained by concatenating the aggregated features from all layers, providing a comprehensive representation of the entire graph, as shown in Eq. 5:

(5) $$h_{G}=\text{concatenate}\;\left(h_{G}^{\left(0\right)},h_{G}^{\left(1\right)},\ldots ,h_{G}^{\left(K\right)}\right),$$

The design of GIN is motivated by its ability to approximate the WL graph isomorphism test, a powerful method for graph comparison. By leveraging an injective aggregation function, GIN is theoretically capable of distinguishing non-isomorphic graphs under certain conditions, making it as expressive as the WL test. The general form of the node update rule can be described as follows:

(6) $$h_{v}^{\left(k\right)}=\phi \left(h_{v}^{\left(k-1\right)},f\left(\left\{h_{u}^{\left(k-1\right)}:u\in 𝒩(v)\right\}\right)\right),$$

In Eq. 6, *f* is a function that aggregates the multiset of neighboring node features, and *ϕ* is an injective function that updates the node features. This formulation ensures that the learned node embeddings are unique for different graph structures, thereby enhancing the model’s discriminative capability.

In the context of the WL test, GIN utilizes an injective hash function *g* to iteratively update the node labels *l_v_* based on the labels of neighboring nodes, as shown in Eq. 7:

(7) $$l_{v}^{\left(k\right)}=g\left(l_{v}^{\left(k-1\right)},\left\{l_{u}^{\left(k-1\right)}:u\in 𝒩(v)\right\}\right),$$

This iterative process resembles the neighborhood aggregation scheme in GIN, where the node features are updated based on the features of adjacent nodes. The expressive power of GIN stems from its ability to learn injective mappings that mirror the behavior of the WL test, enabling it to effectively differentiate complex graph structures [17, 18].

Overall, GIN’s architecture is designed to capture both local and global graph properties, making it particularly well-suited for tasks like graph classification. It has been successfully applied to various domains, including social network analysis, molecular graph classification, and, in this study, the analysis of functional brain networks derived from fMRI data. By leveraging the expressive capabilities of GIN, we can learn meaningful graph embeddings that capture the intrinsic connectivity patterns of the brain, facilitating accurate classification and prediction tasks.

### 3.5 Heat Kernel Diffused Multimodal Graph Isomorphism Network (HKD-MGIN)

The HKD-MGIN is a novel framework designed to analyze multimodal fMRI data by integrating heat kernel diffusion with Graph Isomorphism Networks (GINs). This approach addresses the challenges of multimodal data integration while enhancing the interpretability of functional connectivity by combining principles from diffusion geometry and graph-based deep learning.

The HKD-MGIN framework applies heat kernel diffusion to model pairwise relationships between brain regions, capturing both local and global topological properties of functional brain networks. These diffusion-based representations are then used as input to GINs, which learn meaningful embeddings by modeling complex dependencies within the connectivity graphs. This design enables simultaneous analysis of multiple fMRI modalities or conditions while preserving the underlying geometric structure of brain networks.

Let *M* denote the number of fMRI modalities, with each represented as a graph ${𝒢}^{\left(m\right)}=(𝒱,{ℰ}^{\left(m\right)})$, where 𝒱 is the shared set of nodes (brain regions) and ${ℰ}^{\left(m\right)}$ captures modality-specific functional connections. For each modality *m*, the corresponding adjacency matrix **A**^(*m*)^ is transformed into a heat-diffused kernel matrix using

$$K^{\left(m\right)}=\text{exp}\left(-\beta L^{\left(m\right)}\right),$$

where **L**^(*m*)^ is the graph Laplacian of modality *m*, and *β* is the diffusion parameter that controls the scale of smoothing.

GIN layers are then applied to these diffused graphs to iteratively update node representations. The update rule at the *t*-th layer is given by

(8) $$h_{i}^{\left(t+1\right)}=\sigma \left(W^{\left(t\right)}\cdot \text{Aggregate}\left(\left\{h_{i}^{\left(t\right)}\right\}\cup \left\{h_{j}^{\left(t\right)}:j\in 𝒩(i)\right\}\right)\right),$$

where **W**^(*t*)^ is a learnable weight matrix, *σ* is a non-linear activation function, and Aggregate is a permutation-invariant neighborhood aggregation function. The heat kernel provides a smooth weighting scheme for this aggregation, preserving properties of brain connectivity.

The overall training objective includes a reconstruction loss to maintain fidelity of the heat kernel representation and a supervised loss for downstream tasks, such as age prediction or cognitive classification. The total loss is defined as

(9) $${ℒ}_{\text{HKD-MGIN}}=\overset{M}{\underset{m=1}{\sum }}{\left\|K^{\left(m\right)}-A^{\left(m\right)}\right\|}_{F}^{2}+\lambda \overset{N}{\underset{i=1}{\sum }}\text{Loss}\left(y_{i},{\hat{y}}_{i}\right),$$

where *N* is the number of subjects, **y***_i_* and ${\hat{y}}_{i}$ are the true and predicted labels respectively, and *λ* is a regularization coefficient that balances the reconstruction and predictive losses. Here, the Frobenius reconstruction term ${\left\|K^{\left(m\right)}-A^{\left(m\right)}\right\|}_{F}^{2}$ is not intended to enforce exact equivalence between the diffused kernel *K^(m)^* and the original adjacency matrix *A^(m)^*. Instead, it serves to preserve essential structural information from the original brain graph while preventing the diffusion process from over-smoothing and discarding biologically meaningful connectivity. The regularization parameter *λ* balances this trade-off between structural fidelity and diffusion-driven smoothing, ensuring that the model retains neurobiologically relevant patterns while still benefiting from the denoising properties of diffusion.

HKD-MGIN is particularly suited for analyzing fMRI datasets such as task-based and resting-state fMRI. The framework can uncover consistent and modality-specific patterns of connectivity, offering insights into neurodevelopment, cognition, and potential biomarkers for neurological and psychiatric disorders. The use of heat diffusion enhances interpretability by emphasizing biologically meaningful patterns of signal propagation within the brain network.

### 3.6 GNNExplainer for model interpretability

To improve model interpretability and identify key functional connectivity subnetworks relevant to age prediction, we employed GNNExplainer—a model-agnostic technique for generating explanations in graph neural networks (GNNs) [19]. This approach enabled consistent identification of the most influential node features contributing to prediction outcomes. GNNExplainer selects a subset of features *X_FS_* and learns a feature mask *F*, which is applied to nodes within an explanatory subgraph *G_s_*.

The explanation *(G_s_ , X_s_)* is optimized to maximize a mutual information objective between *G_s_* and *F*, combining structural and feature-based information to explain the model’s prediction $\hat{y}$ for a given node *v* [19]. Optimization involves several hyperparameters, including prediction loss, feature size and element losses, population size and element losses, weight decay, number of epochs, and learning rate, with values specified in Table 2.

The loss functions used by GNNExplainer guide the identification of salient features and structures by penalizing deviations from the original graph. Prediction loss, typically binary cross-entropy, quantifies classification accuracy. Feature size and element losses (mean squared error) measure perturbations in node features. Population size and element losses assess changes in graph structure, including node count and edge weights. Through iterative minimization of these losses, GNNExplainer highlights critical features and subnetworks underlying the model’s predictions [19].

### 3.7 Experimental design

In this study, the dataset was randomly divided into training and testing subsets in proportions of 80% and 20%, respectively. The training set was used to train the model. Model performance was evaluated using root mean square error (RMSE), mean absolute error (MAE), and the correlation coefficient, along with their respective standard deviation (std) values for age prediction. To reduce sampling bias and evaluate model robustness, bootstrap analysis was performed by conducting 10 repeated experiments. Each iteration involved splitting the dataset, training the model, and testing its performance. The outcomes of these repeated experiments were used to compare the performance of our proposed model with other methods. Statistical significance was assessed using pairwise t-tests, and p-values were reported to indicate any significant improvements.

To optimize the performance of the HKD-MGIN model, hyperparameters were fine-tuned using the random search method on the validation sets [20]. As shown in Table 3, the hyperparameters for the experiments included the learning rate, optimizer, number of epochs, weight decay, fMRI paradigms, and the predictive task, with respective values of Adam, 3000, 0.2, emoid/nback, age, and sex. The graph embedding process employed two-layer GIN as described in Eq. 1. The ReLU activation function was utilized within the two-layer HKD-GIN model to integrate graph embeddings derived from the two modalities.

The HKD-MGIN model was applied to working memory and emotion task fMRI data, and its performance was compared with other models (Table 4). The results, including RMSE and MSE along with their corresponding p-values, are summarized in Table 5. Following the evaluation of the model’s performance, we conducted age prediction across the five stages of adolescence to assess whether the proposed framework effectively leveraged functional connectivity as brain fingerprints.

## 4 Results

To optimize the performance of the HKD-MGIN model, hyperparameters were systematically fine-tuned using the random search method on dedicated validation sets [20]. The selected hyperparameters included the learning rate, optimizer, number of epochs, weight decay, fMRI paradigms, and the predictive task. The final values assigned to these parameters were as follows: a learning rate of 1 × 10^−5^, Adam optimizer, 3000 epochs, weight decay of 0.2, and emoid/nback for the fMRI paradigms, with the predictive task centered on age prediction. For graph embedding, a two-layer Graph Isomorphism Network architecture was utilized. The activation layers within the HKD-MGIN model employed the ReLU function, facilitating the effective integration of graph embeddings derived from the two modalities.

A comprehensive evaluation of age prediction and sex classification was conducted using a rigorous 10-fold cross-validation procedure, independently repeated 10 times on the entire dataset. Statistical significance of the classification performance of the HKD-MGIN model, compared to competing approaches, was assessed by calculating p-values through a t-test on the results from repeated experiments. The findings demonstrated that integrating two fMRI paradigms, specifically emoid and nback, led to superior performance relative to using a single paradigm, highlighting the advantages of multi-paradigm fMRI data integration.

To interpret the model, we employed GNNExplainer to identify the critical subnetworks by extracting the top 5% of common functional connections. Both working memory and emotion task fMRI data were analyzed in this investigation. The functional networks were categorized using the following abbreviations: sensorimotor (SM Hand and SM Mouth), visual (VIS), default mode network (DMN), fronto-parietal task control (FRNT), cerebellum (CB), and salience network (SAL). The quantity of common functional connections varied across the five stages of adolescence. Specific regions of interest (ROIs) associated with the most influential connections, comprising the top 5%, were identified for age prediction during adolescence. Figures 6 and 7 illustrates the key intra- and inter-network connections across different stages of adolescence.

### 4.1 Comparison of performance with alternative methods

**Fig. 2:**
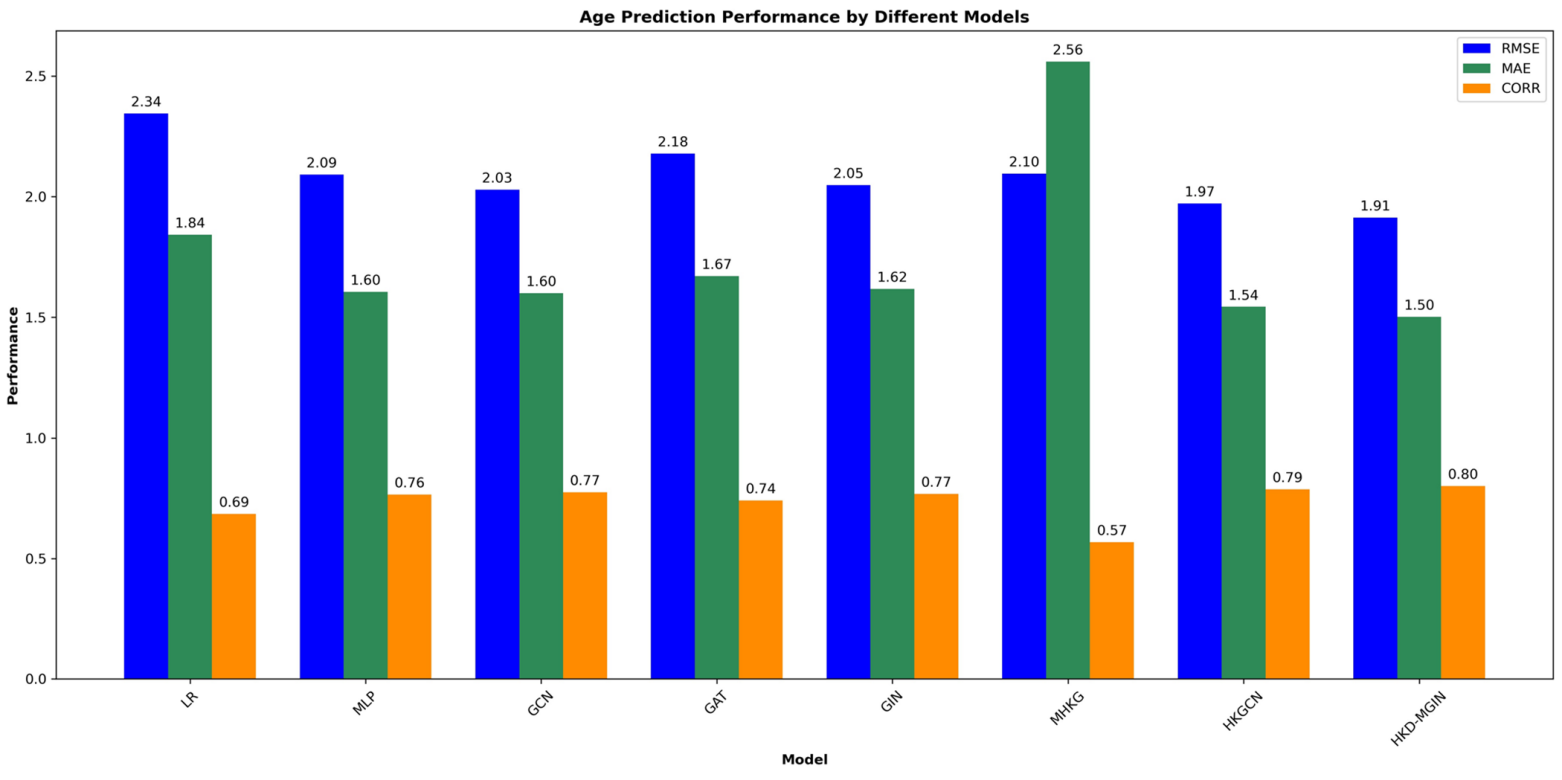
Age prediction performance comparison. Box plots show RMSE, MAE, and correlation coefficients for eight models evaluated using 10-fold cross-validation (repeated 10 times). HKD-MGIN achieved RMSE = 1.91 ± 0.18, MAE = 1.50 ± 0.16, r = 0.80 ± 0.05. Statistical significance assessed using pairwise t-tests

**Fig. 3:**
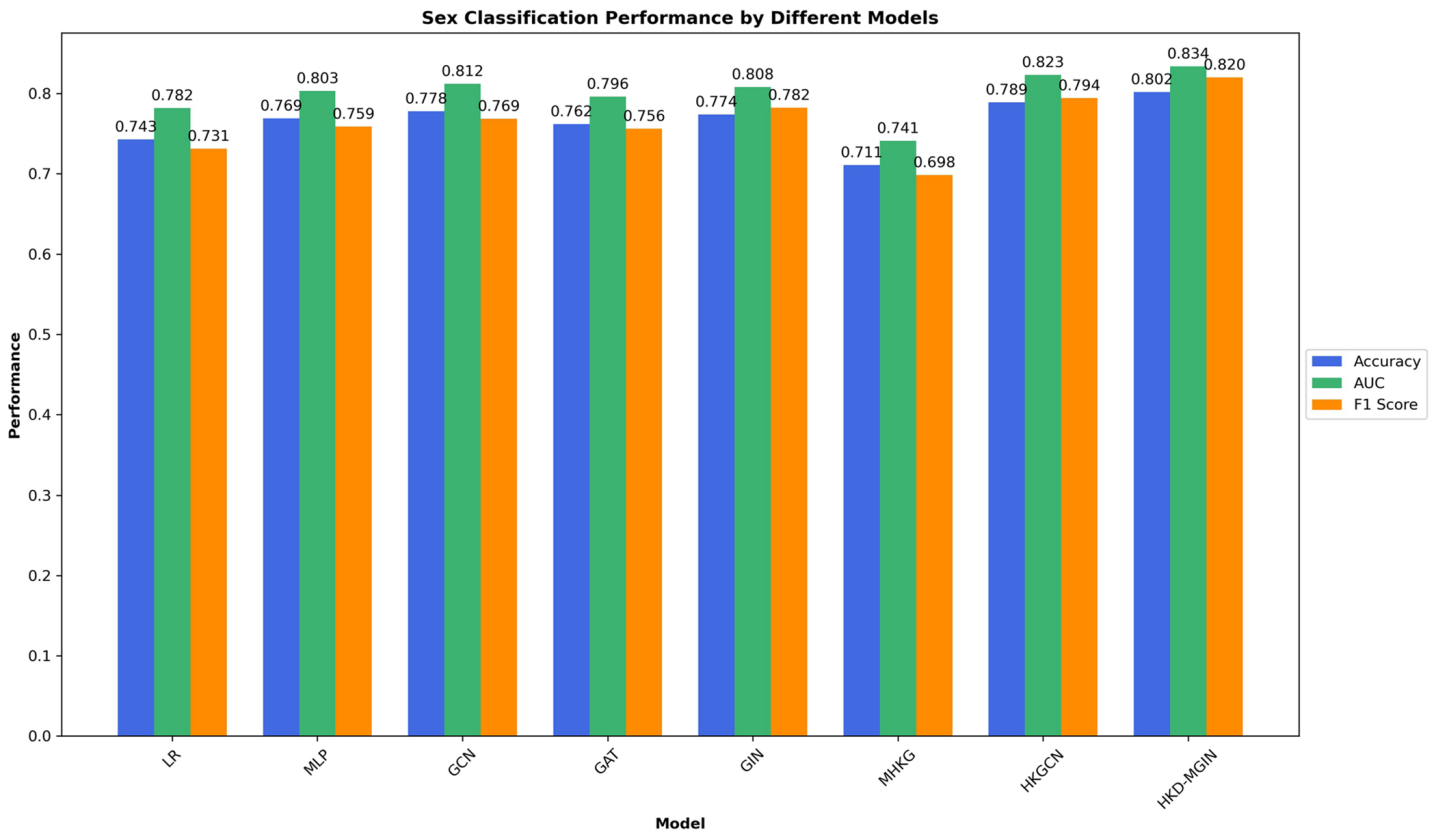
Sex classification performance of different models. Accuracy, AUC, and F1-scores for all models using 10-fold cross-validation. HKD-MGIN performance: accuracy = 0.802 ± 0.015, AUC = 0.834 ± 0.017, F1 = 0.820 ± 0.051

f The results obtained from our proposed model were compared with those of other approaches, and the p-values of pairwise t-tests were reported to demonstrate the statistically significant improvement achieved by our approach. Specifically, we compared HKD-MGIN with several other methods, including:

- **Linear Regression (LR)**: A straightforward statistical technique used as a baseline for age prediction and sex classification. We used the vectorized upper triangular functional connectivity matrix as input to minimize thenumber of parameters.
- **Multi-Layer Perceptron (MLP)**: A fully connected neural network composed of multiple dense layers with non-linear activations. Similar to LR, the upper triangular connectivity matrix was used as input, and L2 regularization and dropout were applied to prevent overfitting.
- **Graph Convolutional Network (GCN)** [21]: A GNN that employs convolutional operations over graph-structured data. The model aggregates information from neighboring nodes to learn both local and global representations. We adopted the standard GCN architecture for our experiments.
- **Graph Attention Network (GAT)** [22]: A GNN that integrates attention mechanisms into the message-passing process, allowing nodes to assign varying importance to their neighbors, which enhances the model’s ability to capture complex relational patterns.
- **Graph Isomorphism Network (GIN)** [17]: A GNN that improves graph-level representation learning through injective aggregation functions. It enhances the model’s ability to distinguish between different graph structures effectively.
- **Multi-Scale Heat Kernel GNN (MHKG)** [23]: An advanced GNN that applies heat kernel diffusion across multiple feature modalities to capture both intra- and inter-modal dependencies in multimodal brain connectivity data.
- **Heat Kernel Graph Convolutional Network (HKGCN)** [24]: A GCN variant that incorporates heat kernel diffusion to better model both local and global relationships within unimodal connectivity data.
- **Heat Kernel Diffused Multimodal Graph Isomorphism Network (HKD-MGIN)**: Our proposed model, which leverages heat kernel diffusion and graph isomorphism principles for learning on multimodal functional connectivity data. By effectively capturing short- and long-range dependencies within and across modalities, HKD-MGIN enables improved representation learning and yields superior performance in classification and prediction tasks related to neurodevelopmental analysis (Figs. 2 and 3).

### 4.2 Ablation experiments

In our ablation studies, we investigated the impact of the heat kernel diffusion mechanism and individual modality contributions on the performance of the proposed HKD-MGIN framework. Specifically, we compared the performance of the model with and without the heat kernel module across three modality configurations: emoid only, nback only, and both combined.

The heat kernel diffusion mechanism is designed to capture multi-scale topological information by diffusing signals over the graph structure. Removing this component effectively reduces the model to a baseline GIN that relies solely on the original connectivity structure, without temporal smoothing or multi-scale propagation.

As shown in Table 6, removing the heat kernel diffusion significantly degraded performance across all modality settings. For the combined emoid + nback configuration, the baseline GIN (no diffusion) yielded a RMSE of 2.2093 ± 0.2481, MAE of 1.6814 ± 0.1872, and correlation of 0.7295 ± 0.0587. In contrast, HKD-MGIN with heat kernel diffusion achieved improved performance with a RMSE of 1.9146 ± 0.1963, MAE of 1.4932 ± 0.1574, and correlation of 0.7986 ± 0.0423.

When comparing modality-specific models, we observed that the emoid-only model consistently outperformed the nback-only model in both the baseline and heat-kernel-enhanced configurations. However, the combined use of both modalities yielded the best results, validating the effectiveness of multimodal integration within the HKD-MGIN framework.

### 4.3 Model explanation and biomarker identification

**Fig. 4:**
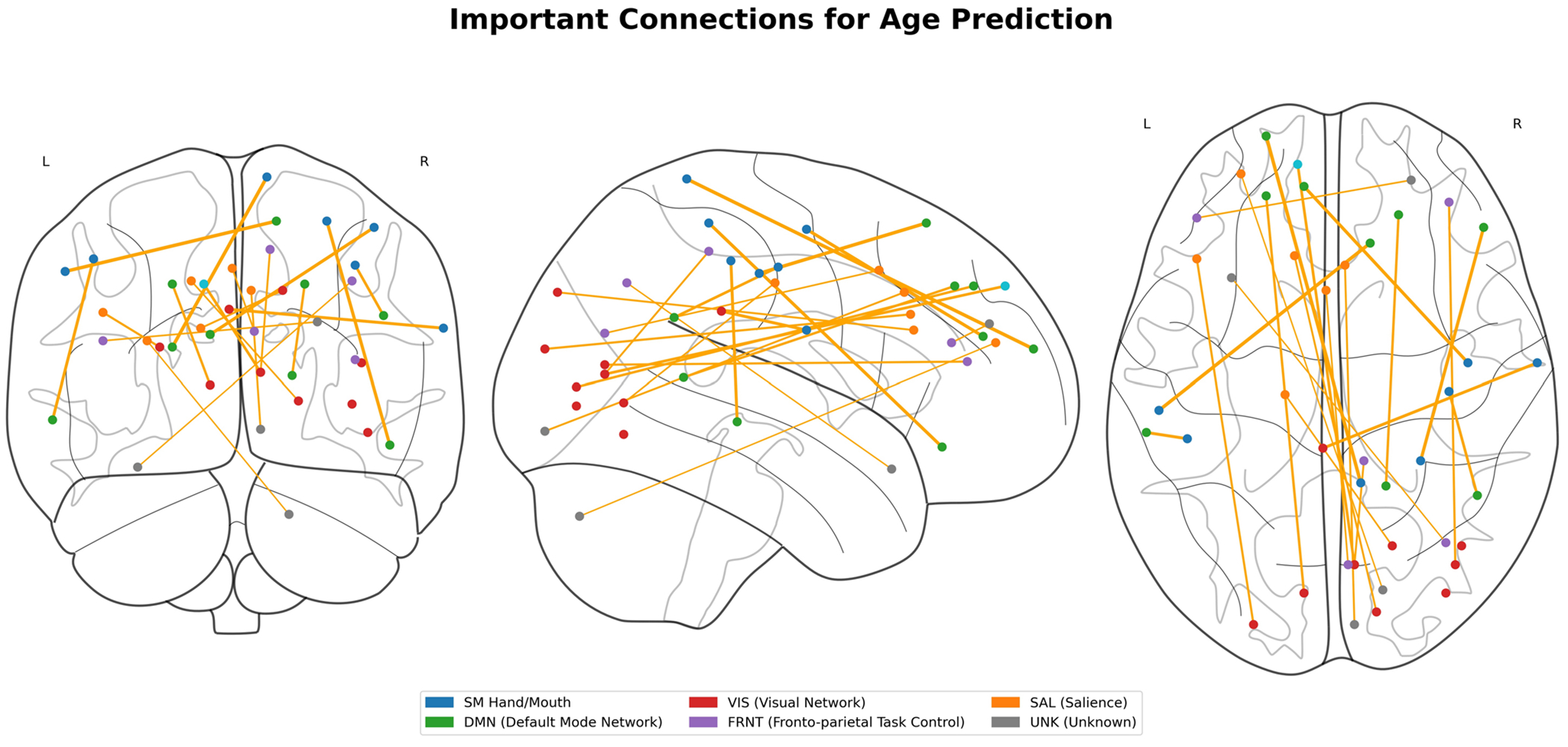
Critical brain regions for age prediction identified by GNNExplainer. Top 0.1% of significant functional connections displayed across axial, coronal, and sagittal views

**Fig. 5:**
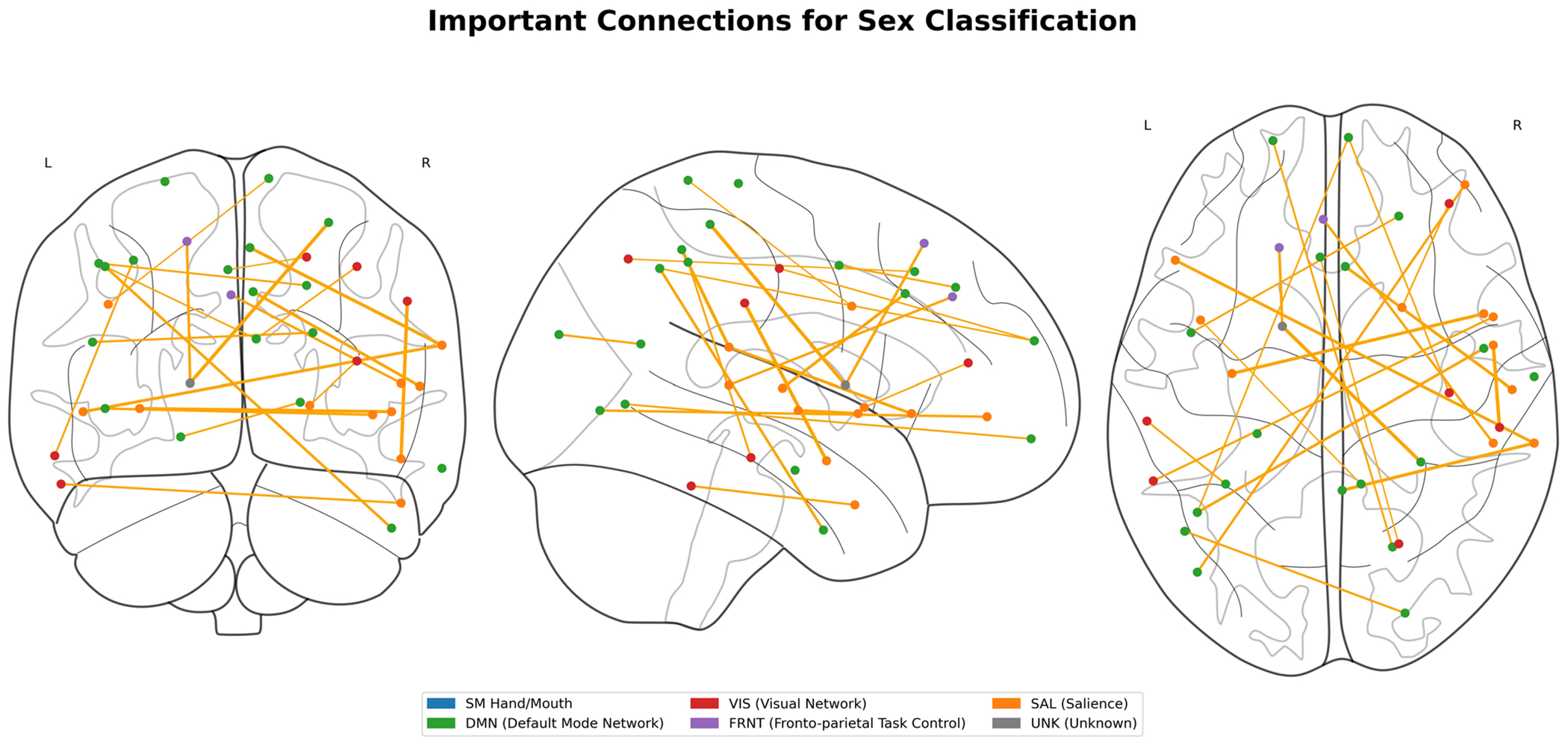
Important connections for sex classification. Sex classification-relevant brain connections identified by GNNExplainer. Top 0.1% of discriminative functional connections shown across three anatomical planes

We employed GNNExplainer to analyze and interpret our model, identifying the top 5% of significant functional connections and highlighting key subnetworks for both working memory and emotion task fMRI data. The functional networks explored encompassed sensorimotor (SM Hand and SM Mouth), visual (VIS), default mode (DMN), frontoparietal task control (FRNT), cerebellum (CB), and salience (SAL). The number of overlapping functional connections varied across the five stages of adolescence. Table 6 presents the top 0.1% of critical connections associated with age prediction during adolescence, while Figs. 4 and 5 provides a visualization of these key regions of interest in axial, coronal, and sagittal planes.

## 5 Discussion

### 5.1 Intra-network connections

A deeper investigation into intra-network connectivity revealed distinct developmental and aging-related changes across different brain networks. These findings shed light on the maturation of cognitive functions and the underlying neural mechanisms that support them.

#### 5.1.1 Memory retrieval (MEM)

The memory retrieval network, involving key regions such as the hippocampus and parahippocampal cortex, undergoes significant alterations across the lifespan, with age-related changes in functional dynamics influencing recall ability. Srokova et al. examined cognitive aging and observed that older adults exhibit a reduced capacity for retrieving detailed episodic memories. Their findings highlighted a phenomenon known as the retrieval-related anterior shift, wherein cortical activity during memory retrieval shifts anteriorly relative to its encoding location [25]. This anterior shift is more pronounced in older individuals and correlates with diminished memory performance, particularly in the parahippocampal place area. This shift is thought to reflect compensatory mechanisms associated with age-related neural dedifferentiation, where the specificity of neural representations declines with aging [26, 27]. Studies using functional MRI have further demonstrated that older adults recruit additional prefrontal and anterior temporal regions during memory retrieval, potentially as a compensatory response to declining hippocampal efficiency [28, 29]. Moreover, age-related alterations in connectivity between the hippocampus and cortical memory networks suggest a reorganization of retrieval mechanisms, possibly driven by reductions in network segregation and increases in dedifferentiation [30, 31]. Longitudinal studies have also shown that memory retrieval impairments in aging are linked to disrupted functional connectivity between the hippocampus and the default mode network (DMN), further supporting the role of network interactions in cognitive decline [32, 33]. Such findings underscore the importance of understanding the interplay between memory-related brain regions and broader network dynamics to develop potential interventions aimed at mitigating age-related memory loss.

#### 5.1.2 Frontroparietal Task Control (FRNT)

The frontoparietal task control network (FRNT) plays a crucial role in cognitive control and executive functioning, particularly in goal-directed behavior and decision-making. Prior research by Dosenbach et al. highlighted developmental shifts in functional connectivity within the FRNT during adolescence, demonstrating that the connectivity strength between the prefrontal cortex and parietal regions increases with age. This strengthening is indicative of ongoing maturation and refinement of network integration, which facilitates enhanced cognitive control processes [34]. Furthermore, Fair et al. reported that adolescence is marked by heightened synchronization and improved functional coordination between these regions, suggesting an increasing efficiency in communication pathways within the FRNT [35]. These findings align with the broader literature indicating that the frontoparietal network undergoes protracted developmental changes that continue into early adulthood, paralleling the maturation of executive functions [36, 37]. FRNT exhibits increasing modular integration with age, reflecting its role in orchestrating cognitive flexibility and adaptive decision-making [38, 39]. Recent evidence also suggests that FRNT connectivity interacts dynamically with sensorimotor and default mode networks during cognitive tasks, strengthening its role in balancing internally and externally directed cognitive processes [40, 41]. These interactions emphasize the importance of cross-network integration in supporting higher-order cognitive abilities that develop progressively through adolescence and into adulthood.

### 5.2 Inter-network connections

**Fig. 6:**
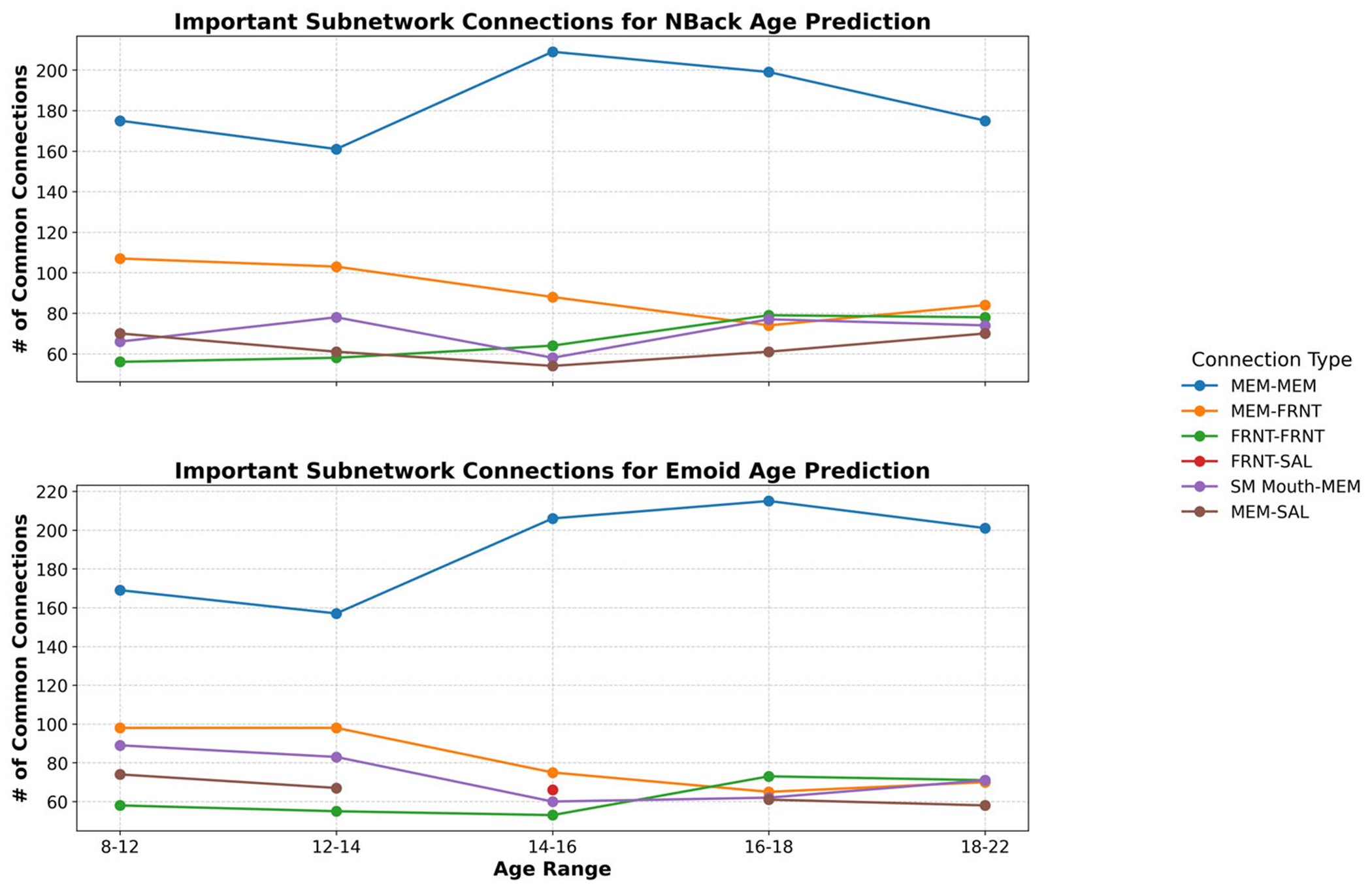
Important subnetwork connections for age prediction during each stage of adolescence. Intra-network and inter-network connections are represented with different colors

**Fig. 7:**
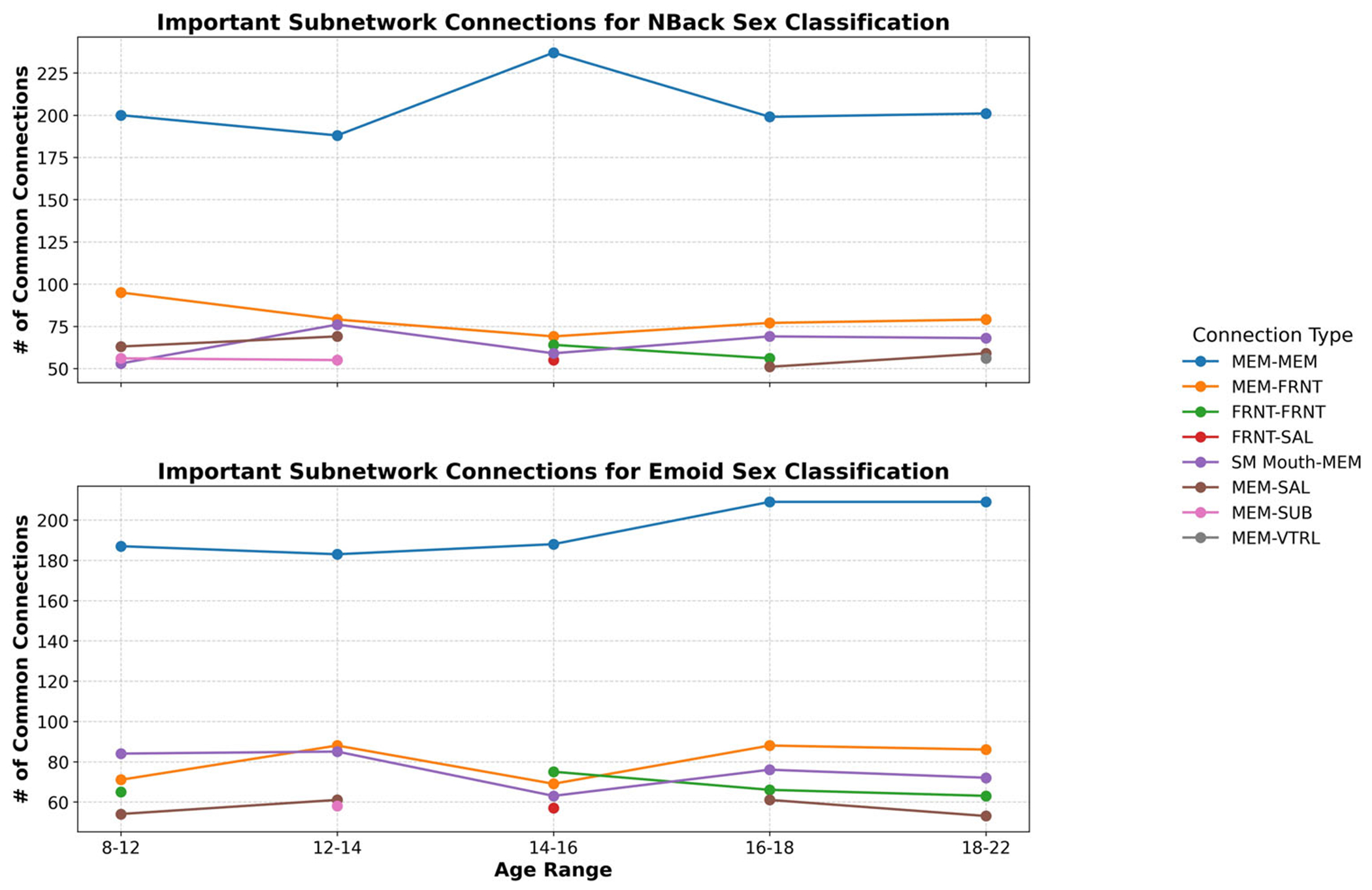
Important subnetwork connections for sex classification during each stage of adolescence. Distinct colors differentiate within-network from between-network functional connections

Beyond intra-network connectivity, the interplay between distinct brain networks also undergoes significant developmental changes, particularly during adolescence. These inter-network interactions are crucial for cognitive maturation, emotional regulation, and decision-making. Our investigation revealed the following key findings (Figs. 6 and 7):

#### 5.2.1 Memory retrieval → frontoparietal task control

Adolescence is a critical period for the maturation of memory retrieval processes and their integration with cognitive control systems, particularly the frontoparietal task control network (FRNT). Episodic memory (EM) retrieval is essential for decision-making, as it allows individuals to draw upon past experiences to guide goal-directed behavior [42]. However, during adolescence, the EM system is still maturing, which may contribute to increased impulsivity and risk-taking behaviors. This aligns with studies indicating that underdeveloped episodic foresight— the ability to use memory for future planning—is linked to heightened engagement in risky behaviors during this developmental stage [43, 44]. In parallel, the FRNT undergoes significant structural and functional refinements, characterized by increased connectivity, enhanced coordination, and improved integration with other neural systems. These changes are vital for the development of executive functions such as working memory, inhibitory control, and goal-directed behavior [44]. Research suggests that as the FRNT strengthens its connections with memory-related regions, adolescents gain better cognitive control over impulsive actions, leading to improved decision-making and a decline in maladaptive risk-taking behaviors [45, 46]. Additionally, network-level integration between memory retrieval and cognitive control mechanisms has been shown to facilitate adaptive learning and behavioral regulation, which become more stable in adulthood [47, 48].

#### 5.2.2 Frontoparietal task control → salience

The interaction between the FRNT and the salience network (SAL) plays a pivotal role in the maturation of self-regulation and attentional control during adolescence. The SAL, primarily anchored in the anterior insula and dorsal anterior cingulate cortex, is responsible for detecting, interpreting, and responding to salient stimuli in the environment. As connectivity between the FRNT and SAL strengthens, adolescents develop a greater capacity for goal-directed behavior and the ability to suppress impulsive responses, marking a transition toward more deliberate and controlled actions [49]. This functional linkage is particularly relevant to adolescent sensitivity to social influences. Adolescents with stronger SAL connectivity are more likely to exhibit prosocial behavior when exposed to positive peer norms but may also be more susceptible to risky behaviors under negative peer influence [50, 51]. This suggests that the interaction between FRNT and SAL networks plays a key role in the evolving balance between self-regulation and social influence during adolescence, with long-term implications for behavioral adaptation and mental health outcomes.

#### 5.2.3 Sensorimotor → memory retrieval

The sensorimotor system, particularly the SM Mouth network, undergoes substantial reorganization during adolescence. A hallmark of this developmental shift is the increasing functional specialization and segregation of sensorimotor networks, wherein connectivity between distinct sensorimotor systems strengthens while within-network connectivity diminishes. At the same time, the memory retrieval (MEM) network is undergoing significant changes, largely influenced by the maturation of the prefrontal cortex. Adolescents progressively shift from relying on perceptual cues for memory retrieval to engaging in more strategic, prefrontal-mediated retrieval processes [52, 53]. The evolving relationship between sensorimotor processing and memory retrieval suggests a dynamic interplay between action-oriented neural systems and cognitive control mechanisms. This connection may support functions such as embodied cognition, where sensorimotor experiences influence memory consolidation and retrieval [54]. Additionally, neuroimaging studies have shown that as individuals age, sensorimotor and cognitive networks become more functionally distinct, reflecting a shift toward more specialized and efficient neural processing [30].

#### 5.2.4 Memory retrieval → salience

Memory retrieval processes are increasingly linked to the salience network (SAL), which facilitates the identification and prioritization of relevant information. This interaction becomes particularly important during adolescence as individuals develop the ability to filter and selectively attend to emotionally and cognitively significant memories. The SAL plays a role in modulating attention toward memory cues based on their relevance, thereby influencing learning and decision-making [49]. As memory retrieval mechanisms mature, the SAL supports the transition from reflexive, stimulus-driven retrieval processes to more controlled and goal-directed memory recall. This transition is critical for emotional regulation and adaptive decision-making, as adolescents rely more on previous experiences to guide future actions. Moreover, research suggests that alterations in SAL connectivity may underlie individual differences in memory-related disorders, such as post-traumatic stress disorder (PTSD) and anxiety, where heightened salience attribution to certain memories leads to maladaptive cognitive patterns [39, 55].

### 5.3 Limitations

The proposed HKD-MGIN model presents certain limitations in its application to fMRI research. First, the current implementation relies on static connectivity measures, such as the Pearson correlation coefficient, to define node features, and cosine similarity is used to compute edge weights. While these methods simplify the modeling process, they may fail to fully capture the temporal and dynamic interactions inherent in brain networks. Future work could address this limitation by incorporating advanced dynamic connectivity metrics and sparsity-inducing constraints, which would enable a more refined representation of edge weights and reduce redundancy in higher-order relationships. Second, the study primarily emphasizes identifying significant functional subnetworks and regions of interest within task-based fMRI data. Although this approach provides valuable insights, it may neglect the broader spectrum of inter-individual variability in brain function. To enhance the model’s generalizability, future research could extend the framework to investigate functional connectivity across diverse cognitive tasks and clinical populations. This expansion would offer a more comprehensive understanding of brain network variability and the model’s applicability across various neuroimaging datasets.

## 6 Conclusion

This study introduced the HKD-MGIN framework for the analysis of multi-paradigm fMRI data. The proposed framework exhibited superior performance in capturing age- and sex-related differences and identifying significant underlying functional networks compared to baseline methods. The model was applied to a cohort of adolescent brain imaging data to predict age and sex across various stages of development. By systematically analyzing shared connections across multi-paradigm data, the model illuminated critical subnetworks associated with age and sex during adolescence. In conclusion, the proposed method offers a useful approach for investigating functional connectivity differences across development and may provide insights relevant to early intervention research. Moreover, the adaptable nature of the framework suggests potential applicability to other imaging modalities and populations, supporting broader use in neuroimaging studies.

Although the absolute numerical improvements achieved by HKD-MGIN may appear modest, in neuroimaging such gains are meaningful due to the high dimensionality and noise of fMRI data. Prior work has shown that even small improvements, when consistent across tasks, indicate greater robustness and reliability [56]. Furthermore, minor performance gains that are coupled with biologically interpretable insights have been emphasized as valuable contributions in adolescent neurodevelopment studies [57]. Similarly, Thapaliya et al. [58] introduced a novel modeling architecture called BrainRGIN to predict various facets of intelligence. Although accuracy improvements were modest, they were meaningful given the high-dimensional, noisy nature of fMRI data, and the model’s identification of specific brain regions associated with intelligence provided valuable neurobiological insights and enhanced interpretability. Thus, the gains achieved by our HKD-MGIN framework are meaningful as they combine consistent predictive improvements with enhanced interpretability.

## Figures and Tables

**Table 1:** Frequently used notation in the HKD-MGIN framework

Notation	Description
**X**	Multimodal data matrix representing fMRI features
**G** = (**V**, **E**)	Graph with vertices *V* (brain regions) and edges *E* (connectivity)
**A**	Adjacency matrix of the graph **G**
**F**	Feature matrix of graph nodes (e.g., regional fMRI measures)
**H**	Heat kernel smoothed node feature matrix
**W**	Weight matrix for the graph isomorphism layers
**Z**	Final embedding matrix after heat kernel diffusion and graph layers
ℒ	Total loss function for optimizing HKD-MGIN
*α* **_ij_**	Attention coefficient for the importance of edge (*i, j*)
**Θ**	Learnable parameters of the HKD-MGIN model
**K**	Number of layers in the graph isomorphism network
**Φ_*t*_**	Heat kernel diffusion matrix at time *t*
$\hat{y}$	Predicted labels
**y**	Ground truth labels for prediction tasks
*λ*	Regularization parameter for the loss function

**Table 2:** Hyperparameters for GNNExplainer

Hyperparameter	Value
Prediction Loss	1
Feature Size Loss	200
Feature Element Loss	20
Population Size Loss	0
Population Element Loss	1000
Weight Decay	0
Training Epochs	100
Learning Rate	5 × 10^−1^

**Table 3:** Hyperparameters for experiments

Learning rate	1e-5
Optimizer	Adam
Epochs	3000
Layer 1 Size	69,696
Layer 2 Size	100
Weight Decay	0.2
fMRI Paradigms	Emoid, Nback
Predictive Task	Age, Sex

**Table 4:** Comparison of age prediction performance by different models

Model	RMSE	P-value	MAE	P-value	CORR	P-value
LR	2.3442 ± 0.2201	0.0480	1.8424 ± 0.1822	0.0701	0.6853 ± 0.0725	0.0045
MLP	2.0915 ± 0.2019	0.0045	1.6048 ± 0.1742	0.2215	0.7645 ± 0.0565	0.0128
GCN	2.0290 ± 0.1853	0.0130	1.5996 ± 0.1588	0.2370	0.7745 ± 0.0559	0.0085
GAT	2.1790 ± 0.2208	0.0380	1.6695 ± 0.1802	0.1845	0.7400 ± 0.0598	0.0219
GIN	2.0472 ± 0.1864	0.0440	1.6172 ± 0.1587	0.0560	0.7684 ± 0.0542	0.0174
MHKG	2.0957 ± 0.0901	0.0646	2.5605 ± 0.0739	0.0640	0.5675 ± 0.0802	0.0151
HKGCN	1.9712 ± 0.2328	0.0271	1.5434 ± 0.2261	0.1178	0.7874 ± 0.0491	0.0318
HKD-MGIN*	**1.9124** ± **0.1812**	–	**1.5012** ± **0.1559**	–	**0.8011** ± **0.0496**	–

* HKD-MGIN denotes our proposed framework. P-values are from pairwise t-tests against HKD-MGIN

Bold entries denote our proposed framework HKD-MGIN

**Table 5:** Comparison of sex classification performance by different models

Model	Accuracy	P-value	AUC	P-value	F1 Score	P-value
LR	0.743 ± 0.019	0.0164	0.782 ± 0.025	0.0164	0.7312 ± 0.0450	0.0153
MLP	0.769 ± 0.021	0.0102	0.803 ± 0.022	0.0102	0.7587 ± 0.0428	0.0125
GCN	0.778 ± 0.018	0.0065	0.812 ± 0.021	0.0065	0.7686 ± 0.0481	0.0112
GAT	0.762 ± 0.020	0.0123	0.796 ± 0.024	0.0123	0.7561 ± 0.0371	0.0064
GIN	0.774 ± 0.017	0.0081	0.808 ± 0.020	0.0081	0.7822 ± 0.0541	0.0180
MHKG	0.711 ± 0.024	0.0018	0.741 ± 0.030	0.0018	0.6984 ± 0.0495	0.0035
HKGCN	0.789 ± 0.016	0.0044	0.823 ± 0.018	0.0044	0.7944 ± 0.0483	0.0073
HKD-MGIN*	**0.802** ± **0.015**	–	**0.834** ± **0.017**	–	**0.8199** ± **0.0514**	–

* HKD-MGIN denotes our framework. P-values are from pairwise t-tests against HKD-MGIN

Bold entries denote our proposed framework HKD-MGIN

**Table 6:** Ablation study results: HKD-MGIN with vs. without heat kernel and different modalities

Model Configuration	Performance Metrics (mean ± std)		
	RMSE	MAE	Corr
Baseline GIN (No Heat Kernel, Emoid Only)	2.0183 ± 0.2156	1.6027 ± 0.1693	0.7682 ± 0.0512
Baseline GIN (No Heat Kernel, Nback Only)	2.1361 ± 0.2298	1.6509 ± 0.1801	0.7511 ± 0.0553
Baseline GIN (No Heat Kernel, Emoid + Nback)	2.2093 ± 0.2481	1.6814 ± 0.1872	0.7295 ± 0.0587
HKD-MGIN (With Heat Kernel, Emoid Only)	1.9754 ± 0.1891	1.5459 ± 0.1607	0.7831 ± 0.0450
HKD-MGIN (With Heat Kernel, Nback Only)	2.1036 ± 0.2412	1.6392 ± 0.1850	0.7523 ± 0.0543
HKD-MGIN (With Heat Kernel, Emoid + Nback)	**1.9146** ± **0.1963**	**1.4932** ± **0.1574**	**0.7986** ± **0.0423**

1 Standard deviation is represented by “std”

Bold entries denote our proposed framework HKD-MGIN

## Data Availability

The data is available in a publicly accessible repository.
